# Personality Change following Internet-Based Cognitive Behavior Therapy for Severe Health Anxiety

**DOI:** 10.1371/journal.pone.0113871

**Published:** 2014-12-01

**Authors:** Erik Hedman, Gerhard Andersson, Nils Lindefors, Petter Gustavsson, Mats Lekander, Christian Rück, Erik Andersson, Brjánn Ljótsson

**Affiliations:** 1 Department of Clinical Neuroscience, Division of Psychiatry, Karolinska Institutet, Stockholm, Sweden; 2 Department of Clinical Neuroscience, Osher Center for Integrative Medicine, Karolinska Institutet, Stockholm, Sweden; 3 Department of Clinical Neuroscience, Division of Psychology, Karolinska Institutet, Stockholm, Sweden; 4 Department of Behavioural Sciences and Learning, Linköping University, Linköping, Sweden; 5 Swedish Institute for Disability Research, Linköping University, Linköping, Sweden; 6 Stress Research Institute, Stockholm University, Stockholm, Sweden; University Hospital of Bellvitge-IDIBELL; CIBER Fisiopatología Obesidad y Nutrición (CIBERObn), Instituto Salud Carlos III; Department of Clinical Sciences, School of Medicine, University of Barcelona, Spain, Spain

## Abstract

Personality traits have traditionally been viewed as stable, but recent studies suggest that they could be affected through psychological treatment. Internet-based cognitive behavior therapy (ICBT) for severe health anxiety (DSM-IV hypochondriasis) has been shown to be effective in reducing health anxiety, but its effect on measures of personality traits has not been investigated. The main aim of this study was to investigate the impact of ICBT on personality traits in the three broad dimensions - neuroticism, extraversion and aggression. We hypothesized that participants in ICBT would reduce their level of neuroticism compared to controls that did not receive the active treatment. No specific predictions were made regarding extraversion and aggression. Data from a randomized controlled trial were used in which participants were allocated to 12 weeks of ICBT (n = 40) or to a basic attention control condition (n = 41). Personality traits were assessed with the Swedish Universities Scales of Personality and the primary outcome of health anxiety was the Health Anxiety Inventory. There was a significant interaction effect of group and time on neuroticism-related scales, indicating larger pre- to post-treatment reductions in the Internet-based CBT group compared to the control condition. Analyses at 6-month follow-up showed that changes were stable. Traits relating to extraversion and aggression were largely unchanged. This study is the first to demonstrate that a brief ICBT intervention for severe health anxiety causes long-term changes in measures of personality traits related to neuroticism. The treatment thus has a broader impact than just reducing health anxiety.

**Trial Registration:**

Clinicaltrials.gov (ID NCT00828152)

## Introduction

Persons with severe health anxiety, in the present study defined as meeting the DSM-IV criteria for hypochondriasis, have a persistent fear of developing serious somatic disease [1]. If untreated, severe health anxiety is chronic for a majority of the affected and leads do functional disability and substantial suffering [2], [3], [4], [5]. In the treatment of severe health anxiety, cognitive behavior therapy (CBT) has been shown to yield large and long-term enduring effects [6], [7], [8], [9]. We recently conducted a randomized controlled trial of therapist guided Internet-based CBT (ICBT) for severe health anxiety which showed that CBT based on exposure and response prevention can lead to large improvements when delivered via the Internet with 80 percent of participants in remission at 6-month follow-up [10]. Internet-based treatments of this type generally entails no real time contact between patient and therapist but relies heavily on extensive self-help texts which the patient gets gradual access to through an Internet-based treatment platform [11]. Communication between patient and therapist is mainly through an email like online messaging system and throughout the treatment the patient is expected to go through the same behavioral change as would be the case in face-to-face treatment. Previous studies investigating ICBT for other disorders have shown that the effects can be on par with those of face-to-face treatment [12], [13].

For at least 2000 years there have been more or less complex models of human personality traits [14], [15], likely stemming from the fact that many behavioral patterns are stable over time but differ between individuals in the same situation [16]. Theorists have suggested different types of personality classifications with varying number of personality dimensions [15]. One personality dimension common to all major classifications is neuroticism, which has been described as “the tendency to experience negative emotions and respond poorly to stress” [14], [15], [17]. Several studies have shown that neuroticism is associated with anxiety and anxiety disorders [18], [19]. The Swedish Universities Scales of Personality (SSP) measures 13 traits that make up three broad personality dimensions - neuroticism, extraversion and aggression [20]. Of specific interest to the present study, the five personality traits included in the neuroticism dimension are psychic and somatic trait anxiety, stress susceptibility, embitterment, and lack of assertiveness.

The assumption that personality traits are stable has recently been questioned and new large-scale longitudinal studies and meta-analyses of previous research have established the changeability of traits [21], [22]. To some extent, this fits with a learning theory perspective which posits that environmental changes could lead to changes in personality traits but that personality would not be meaningful to regard as separate from respondent and operant behaviors [23]. As early as in 1959, Eysenck suggested that exposure-based behavior therapy could be effective in reducing neuroticism while assuming genetically based inter-individual differences of the speed in which conditioned responses are learned and habituation occurs [24]. According to this view, persons with high levels of neuroticism are characterized by fast learning of conditioned anxiety responses and slow habituation when exposed to anxiety-triggering stimuli, which is a similar to Gray's behavioral inhibition system theory of temperament [25]. Thus, changeability of personality traits is consistent with empirical findings as well as with trait and learning theory. The environment as a determinant of trait changes could be regarded as specifically interesting as it is relatively easily to manipulate, at least in comparison to other variables suggested to play a role in trait development, such as genes and neurobiological structures.

When it comes to psychological treatment as a potential environmental determinant of personality change the empirical data is scarce and to our knowledge no previous study has investigated personality change after treatment for severe health anxiety. Looking in other fields than severe health anxiety, a study of long-term psychodynamic therapy showed that neuroticism-related personality traits changed after treatment, i.e. there was a reduction of neuroticism [26]. In that study, the therapies however lasted three years on average and there was no randomization to a control group making attribution of the effect of the personality change to treatment uncertain. In a trial comparing two forms of CBT to treatment as usual for social anxiety disorder, the personality trait harm avoidance, which is similar to neuroticism but based in Cloninger's model of personality [27], was significantly reduced after treatment but there was no difference in change between treatments [28]. In two open trials of pharmacotherapy for obsessive-compulsive disorder and of CBT for bulimia nervosa, respectively, it was also found that harm avoidance was reduced after treatment [29], [30]. In the area of depression, two studies are of high relevance to the present one. Firstly, Tang and co-workers showed that cognitive therapy as well as treatment with SSRI can reduce neuroticism and increase extraversion in depressed patients using a randomized placebo-controlled design [31]. Secondly, in a trial of ICBT for depression participants made significant reductions in the trait harm avoidance, but as in the study on social anxiety disorder, there was no difference in trait changes between treatment conditions thus making it uncertain whether the treatment caused the change in personality [32].

Considering the hypothesized mechanisms and content of exposure-based CBT it would be unlikely that this treatment would have the same impact, if any, on all personality traits. Instead, it is reasonable to assume that the treatment, aimed at making the patient confront fears, would have a larger impact on traits of neuroticism. As stated above, the question of personality change has not been previously addressed in the treatment of severe health anxiety and more knowledge in this regard is important to establish the scope of the impact of the treatment. That is, is the effect of the treatment limited to health anxiety reduction or does it also influence behavioral patterns commonly referred to as personality traits?

The main aim of the present study was to investigate the effect of ICBT for severe health anxiety on personality traits using data from a randomized controlled trial. We hypothesized that participants in ICBT would reduce their level of neuroticism-related traits compared to controls that did not receive active treatment. No specific predictions were made in terms of changes in traits of extraversion- or aggression-related traits.

## Methods

### Design

This study used data from a randomized controlled trial [33] where participants with a principal diagnosis of severe health anxiety were randomly allocated to 12 weeks of ICBT (n = 40) or to a control condition that received no active treatment but had access to discussion forum (n = 41). Participants in the control condition were crossed over to ICBT after 12 weeks. In the trial, assessments of psychiatric symptoms and personality were conducted before treatment (pre-treatment), after treatment (post-treatment), and six months post-treatment (6 MFU). As participants in the control condition were crossed over to treatment after post-treatment, no between group comparisons could be made at 6 MFU. After having received ICBT, participants who initially were allocated to the control condition conducted a second post-treatment assessment (post-treatment 2). In this study, the control group data from post-treatment to post-treatment 2, i.e. measurements directly before and after they received Internet-based CBT, was used for replication. That is, we expected that potential changes in personality in the main analyses would also be found in the control group after it had received treatment. The group that immediately received treatment did not significantly differ from the control group in terms of health anxiety or on demographic variables at baseline. The study was conducted in a university hospital setting in Stockholm, Sweden and all participants provided written informed consent. The trial was approved by the regional ethics review board in Stockholm, registered with Clinicaltrials.gov (ID NCT00828152), and conducted in accordance with the guidelines of the Declaration of Helsinki. Participants provided written informed consent electronically over the Internet and the findings of the main outcome study have been previously reported [33], [34].

### Main inclusion criteria and sample characteristics

The main inclusion criteria were that participants had to: (a) have a principal diagnosis of severe health anxiety, i.e. hypochondriasis, according to DSM-IV [1], (b) agree not to undergo any other psychological treatment for the duration of the study, (c) have no history of psychosis or bipolar disorder, and (d) have a constant dosage two months prior to treatment if on prescribed medication for anxiety or depression and agree to keep dosage constant throughout the study. Table 1 displays the characteristics of the participants at baseline. Participants were recruited through referral or self-referral to the university hospital clinic in Stockholm, Sweden, where the study was conducted. Recruitment was done nationally, i.e. adult individuals in all of Sweden could apply for the study, and a clinical psychologist conducted a structured assessment interview via telephone prior to inclusion to establish whether inclusion criteria were met. The diagnostic instruments used are presented under “Diagnostic assessment” below.

**Table 1:** Description of the participants.

		ICBT	Control condition
		n = 40	n = 41
**Baseline health anxiety**	Mean HAI score (SD)	107.0 (22.0)	106.0 (16.6)
**Baseline depressive symptoms**	Mean MADRS-S score (SD)	12.3 (5.9)	13.7 (7.6)
**Baseline global functioning**	Mean GAF score (SD)	54.8 (3.1)	55.0 (4.3)
**Gender**	Women	28	32
	Men	12	9
**Age**	Mean age (SD	39.3 (9.8)	38.8 (9.5)
	Min-max	25–62	25–69
**Duration of severe health anxiety**	Mean duration, years (SD	20 (13.8)	21.95 (12.4)
**Occupational status**	Working 75–100%	31	32
**Referral**	Self-referral	28	28
	Referred	12	13

Note: ICBT, Internet-based cognitive behavior therapy; HAI, Health Anxiety Inventory; MADRS-S, Montgomery Åsberg Depression Rating Scale Self-rated; GAF, Global Assessment of Functioning.

### Measures

#### Personality

To assess personality traits the Swedish Universities Scales of Personality (SSP) was used [20]. This personality inventory was developed to tap into stable personality dimensions that could be linked to biological markers and psychopathology. The SSP comprises 13 trait scales measured with 91 Likert scale items each rated from 1 (does not apply at all) to 4 (applies completely). The score of each scale is calculated as the average score of the items of the respective scales. Factor-analytic evidence indicates that the 13 scales primarily measure three broad personality dimensions which are neuroticism, extraversion and aggression [20]. Table 1 presents a detailed description of each SSP personality scale. The inventory has been shown to have high internal consistency (Cronbach's *α* = .73–.84 on all scales except the SD scale with α = .59) [20]. Reliability estimates, presented in Table 2, were similar when analyzing SSP data from the present study. The scale was originally developed in Swedish, but is also available in English translation. The SSP and its predecessor the Karolinska Scales of Personality (KSP) have demonstrated high predictive value in terms of associations with occupational impairment, family functioning and health satisfaction [35], and have also been validated against objective biological data such as Dopamine-2 receptor density [36], [37].

**Table 2:** Description of the Swedish Scales of Personality including reliability estimates.

SSP Scale trait	Personality factor	Reliability (α)	Item example
**Psychic trait anxiety (PSTA)**	**Neuroticism**	.71	I'm the kind of person who is excessively sensitive and easily hurt
**Somatic trait anxiety (STA)**	**Neuroticism**	.74	My body often feels stiff and tense
**Stress susceptibility (SS)**	**Neuroticism**	.75	I get tired and hurried too easily
**Lack of assertiveness (LA)**	**Neuroticism**	.82	Although I know I'm right I often have great difficulty getting my point across
**Embitterment (E)**	**Neuroticism**	.78	I have often got into trouble even when it was not my fault
**Adventure seeking (AS)**	**Extraversion**	.89	I have an unusually great need for change
**Detachment (D)**	**Extraversion**	.80	I feel best when I keep people at a certain distance
**Impulsiveness (I)**	**Extraversion**	.78	I have the tendency to act on the spur of the moment without really thinking ahead
**Social desirability (SD)**	**Aggression**	.43	No matter whom I'm talking to, I'm always polite and courteous
**Verbal trait aggression (VTA)**	**Aggression**	.77	When I get angry I often express myself ironically or sarcastically
**Physical trait aggression (PHTA)**	**Aggression**	.87	If someone hits me, I hit back
**Trait irritability (TI)**	**Aggression**	.79	I don't have so much patience
**Misstrust (M)**	**Aggression and neuroticism**	.85	I tend to be on guard with people who are somewhat more friendly than I expected

Note: Each scale comprises 7 items and has a 1–4 scale range; SSP, Swedish Scales of Personality; reliability coefficients are baseline Cronbach's αs from the present study.

#### Health Anxiety

The primary outcome measure of health anxiety was the Health Anxiety Inventory [HAI; 38]. The HAI consists of 64 items scored from 0 to 3 which yields an overall score ranging from 0 to 192. The HAI has demonstrated high internal consistency (Cronbach's *α* = .95) and good test-retest reliability (*r* = .90) [38]. The internal consistency was high also in the present study (Cronbach's *α* = .93).

#### Diagnostic assessment

To establish whether participants met diagnostic criteria for severe health anxiety and other Axis I disorders, we used the Health Anxiety Interview [39] and the Mini International Neuropsychiatric Interview [MINI; 40]. Global assessment of functioning (GAF) was assessed by the interviewer using the GAF-scale [1].

### Randomization and procedures

Randomization was conducted using a true random number service (www.random.org) in a 1∶1 ratio with no restriction or masking. Diagnostic interviews were conducted by four clinical psychologists via telephone, which has been shown to be a reliable administration format for diagnostic assessment and self-report measures [41], [42]. The SSP and the HAI were administered via the Internet, which is an administration format that is as reliable and valid as paper-and-pencil assessment [43], [44]. In the main outcome study [33], other measures, such as the Montgomery Åsberg Depression Rating Scale Self-rated [45], were also used. A more detailed description of the procedures of the trial has been previously reported [33].

### Internet-based CBT

The main component of the treatment were 12 modules comprising extensive self-help texts that were designed to provide the patient with the same knowledge and to promote the same behavior change as would be the case had the treatment been administered face-to-face [33]. The 12 modules were made accessible through an Internet-based treatment platform and throughout the treatment patients had contact with the same therapist via a secure online contact system resembling email. Participants were granted gradual access to the modules by their therapist and each module was devoted to a specific theme and included homework exercises. The duration of the treatment was 12 weeks.

The treatment was based on a CBT model for health anxiety, emphasizing the role of avoidance and safety behaviors, internal focus, and interpretations of bodily sensations as signs of serious illness as maintaining factors for hypochondriasis [39], [46]. The main intervention was exposure to health anxiety-related events in combination with response prevention [47]. The treatment also included a form of mindfulness training, which comprised exercises with the aim of enhancing the patients' ability to experience bodily sensations without trying to control them or seek reassurance. Benefits of mindfulness training in cognitive therapy for severe health anxiety have been demonstrated in recent studies [48], [49]. In the present study mindfulness was not used as a stand-alone intervention but was employed specifically to optimize effectiveness of exposure and response prevention exercises. As described by Treanor [50], mindfulness training could facilitate extinction learning during exposure through increasing awareness of multiple conditioned triggers of anxiety.

The treatment protocol was developed by our research group and has been shown to be effective in reducing health anxiety both in face-to-face format and when delivered via the Internet [51]. Therapists conducting the treatments were four clinical psychologists who spent 9 minutes (*SD* = 5.6) weekly per patient on average. As a general rule, therapists were instructed to have at least one contact per week with the patients. As reported in the main outcome study [33], the treatment was effective in reducing health anxiety yielding large between-group effect sizes at post-treatment (Cohen's *d* = 1.62; 95% CI = 1.10–2.10). At post-treatment, 27 participants (67.5%) in the Internet-based CBT group no longer met diagnostic criteria for severe health anxiety which was significantly more than the two participants (4.9%) in the control group who did not meet diagnostic criteria at the same time (χ^2^_(1)_ = 34.55, *p*<.001).

### Control condition

The control condition had access to an online discussion forum where participants could communicate with other trial participants in the control condition. This was a form of basic attention control condition and participants did not receive active treatment in terms of getting access to text modules or going through a systematic behavioral change.

### Statistical analysis

Statistical analyses were conducted using SPSS version 22.0 (IBM Corp., Armonk, NY). Data were analyzed on an intent-to-treat basis meaning that all participants were analyzed in accordance to their allocation status. The main between-group comparisons were conducted within a linear mixed effects models framework for repeated data, modeling group and time (pre-to post-treatment) as fixed effects and individual differences in baseline levels and trajectories, i.e. intercept and slope, as random effects. To assess whether there was a main effect of group on each broad personality factor, i.e. aggregated neuroticism scales, extraversion scales and aggression scales, MANOVAs were conducted. Investigation of potential baseline between-group differences on SSP trait scales was done using independent t-tests. Within-group changes were analyzed with *t*-tests and associations of health anxiety and personality traits were carried out using bivariate zero-order correlations. Effect sizes were calculated using Cohen's *d* based on pooled standard deviations. Throughout, an alpha-level of.05 was used.

## Results

### Attrition and adherence

There was no data loss at pre-treatment, post-treatment or 6-month follow-up. At post-treatment 2, i.e. after the control group had received Internet-based CBT, 39 of 41 (95%) of participants completed assessments. On average, participants completed 8.1 modules (*SD* = 3.9).

### Effects of Internet-based CBT

#### Impact of Internet-based CBT on personality traits

**Figure 1:**
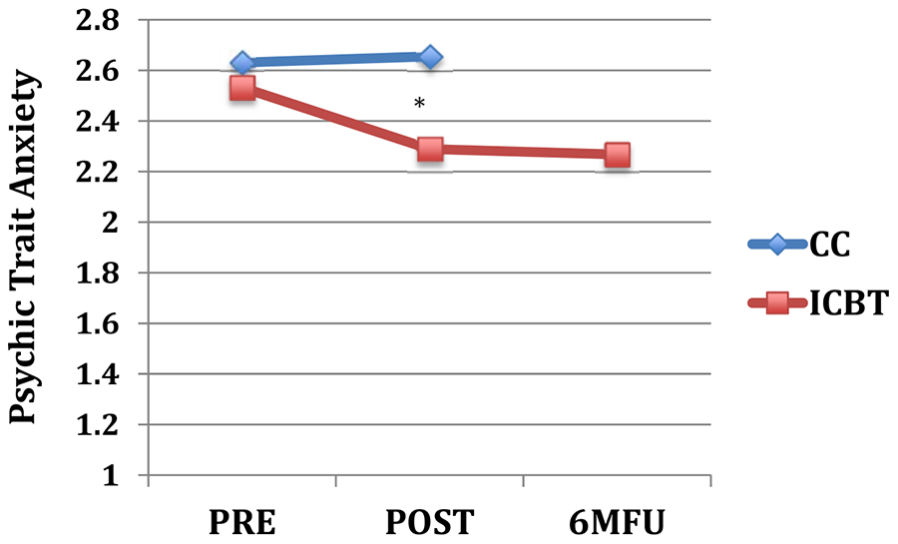
Psychic Trait Anxiety across assessment points. Note: * = *p*<.05 for interaction effect of group and time (pre-to-post); Pre, pre-treatment; Post, post-treatment; 6 MFU, 6-month follow-up; CC, control condition; ICBT, Internet-based CBT.

Table 3 displays means and SDs on personality trait scales of the SSP at each assessment point and Table 4 inter-correlations of the SSP scales and the HAI. Analyses with t-tests showed no significant between-group differences on any of the SSP trait scales at baseline (t_(79)_ = −1.5–0.2, *p* = .12–.96). MANOVA analysis revealed a significant main effect of group on neuroticism change scores using all six neuroticism scales as dependent variables (*F*_(6)_ = 3.2, *p* = .007). There was no significant effect on group from MANOVAs using extraversion scales as dependent variables (*F*_(3)_ = 0.4, *p* = .78) or when using aggression scales as dependent variables (*F*_(5)_ = 1.6, *p* = .17). Subsequent mixed effects models analyses showed a significant interaction effect of group and time on neuroticism-related scales Psychic Trait Anxiety, Somatic Trait Anxiety, Stress Susceptibility, and Embitterment, indicating larger pre- to post-treatment reductions in the ICBT group compared to the control condition (*F*_(1, 79)_ = 7.2–14.0, *p* = .0.00–.019). There was also a significant interaction effect on the trait scale Mistrust indicating larger pre- to post-treatment reductions in the Internet-based CBT group than in the control condition (*F*_(1, 79)_ = 5.4, *p* = .023). There were no differential between-group change patterns on the other personality traits, i.e. Lack of Assertiveness, Adventure Seeking, Detachment, Social Desirability, Verbal and Physical Trait aggression, and Trait Irritability (*F*_(1, 79)_ = 0.0–3.2, *p* = .075–.997). As shown in Table 3, between group effect sizes at post-treatment were moderate to large on neuroticism-related scales and small to moderate on extraversion and aggression trait scales. Figures 1 through 4 displays the change over time on the neuroticism-related scales with significant interaction effects of time and group.

**Figure 2:**
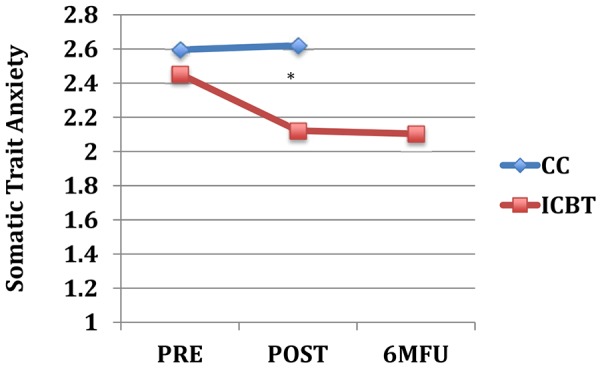
Somatic Trait Anxiety across assessment points. Note: * = *p*<.05 for interaction effect of group and time (pre-to-post); Pre, pre-treatment; Post, post-treatment; 6 MFU, 6-month follow-up; CC, control condition; ICBT, Internet-based CBT.

**Figure 3:**
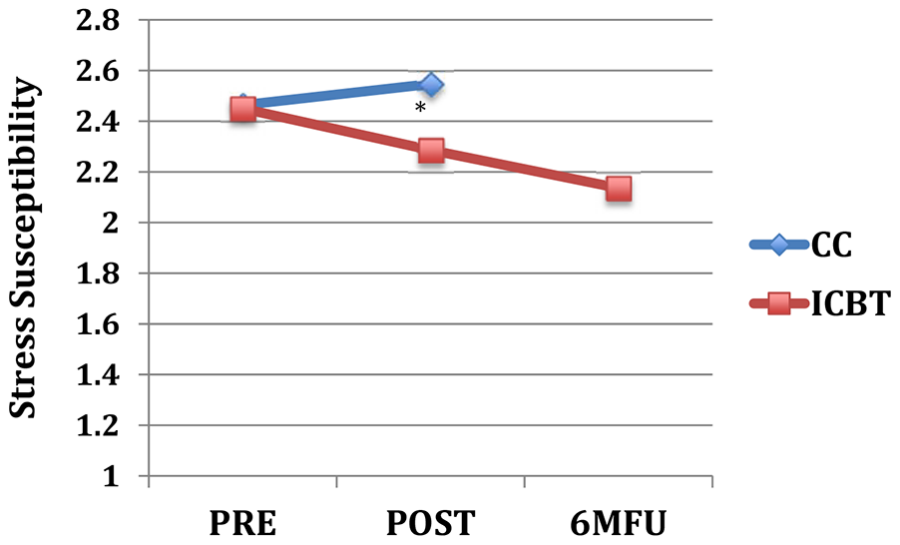
Stress Susceptibility across assessment points. Note: * = *p*<.05 for interaction effect of group and time (pre-to-post); Pre, pre-treatment; Post, post-treatment; 6 MFU, 6-month follow-up; CC, control condition; ICBT, Internet-based CBT.

**Figure 4:**
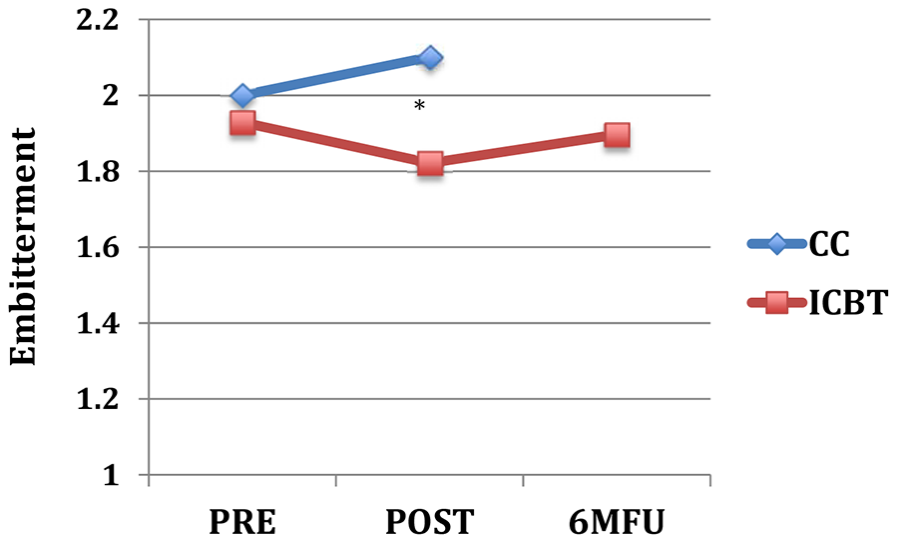
Embitterment across assessment points. Note: * = *p*<.05 for interaction effect of group and time (pre-to-post); Pre, pre-treatment; Post, post-treatment; 6 MFU, 6-month follow-up; CC, control condition; ICBT, Internet-based CBT.

**Table 3:** Personality trait estimates and their effect sizes across assessment points.

						Effect size	Effect size	Effect size
SSP trait	Group	Pre	Post	Post 2	6 MFU	Between	Within	Within
		M (SD)	M (SD)	M (SD)	M (SD)	Post (95% CI)	Pre-Post (95%CI)	Pre-FU (95%CI)
**Neuroticism**								
Psychic trait anxiety	ICBT	2.53 (0.51)	2.29 (0.50)		2.27 (0.60)	0.64 (0.19–1.08)	0.48 (0.25–0.71)	0.47 (0.19–0.75)
	CC	2.63 (0.60)	2.66 (0.64)	2.34 (0.66)			−0.05 (−0.27–0.19)	
Somatic trait anxiety	ICBT	2.45 (0.62)	2.12 (0.60)		2.10 (0.69)	0.82 (0.36–1.26)	0.55 (0.31–0.78)	0.53 (0.31–0.74)
	CC	2.60 (0.54)	2.62 (0.62)	2.26 (0.60)			−0.04 (−0.28–0.20)	
Stress susceptibility	ICBT	2.45 (0.46)	2.29 (0.53)		2.14 (0.52)	0.48 (0.03–0.91)	0.33 (0.03–0.62)	0.63 (0.40–0.86)
	CC	2.46 (0.52)	2.55 (0.56)	2.34 (0.64)			−0.15 (−0.37–0.06)	
Lack of assertiveness	ICBT	2.16 (0.50)	2.02 (0.53)		2.00 (0.55)	0.45 (0.00–0.88)	0.28 (0.02–0.54)	0.29 (0.07–0.52)
	CC	2.36 (0.68)	2.28 (0.63)	2.18 (0.62)			0.12 (−0.07–0.31)	
Embitterment	ICBT	1.93 (0.47)	1.82 (0.49)		1.90 (0.56)	0.51 (0.06–0.94)	0.22 (0.00–0.45)	0.06 (−0.14–0.26)
	CC	2.00 (0.56)	2.10 (0.61)	1.88 (0.50)			−0.17 (−0.41–0.07)	
**Extraversion**								
Adventure seeking	ICBT	2.28 (0.55)	2.24 (0.64)		2.28 (0.61)	0.25 (−0.19–0.69)	0.06 (−0.13–0.26)	0.00 (−0.44–0.44)
	CC	2.38 (0.73)	2.40 (0.63)	2.31 (0.71)			−0.03 (−0.20–0.15)	
Detachment	ICBT	1.81 (0.49)	1.75 (0.48)		1.76 (0.51)	0.46 (0.01–0.90)	0.11 (−0.09–0.32)	0.09 (−0.12–0.30)
	CC	1.97 (0.42)	1.97 (0.48)	1.95 (0.36)			−0.02 (−0.28–0.25)	
Impulsiveness	ICBT	2.28 (0.54)	2.24 (0.53)		2.36 (0.59)	−0.18 (−0.26–0.61)	0.07 (−0.13–0.28)	−0.14 (−0.36–0.09)
	CC	2.27 (0.58)	2.26 (0.60)	2.20 (0.64)			0.02 (−0.15–0.20)	
**Aggression**								
Social desirability	ICBT	2.72 (0.38)	2.81 (0.34)		2.79 (0.34)	−0.27 (−0.70–0.17)	−0.25 (−0.57–0.08)	−0.19 (−0.47–0.08)
	CC	2.70 (0.34)	2.71 (0.40)	2.71 (0.38)			−0.03 (−0.29–0.24)	
Verbal trait aggression	ICBT	2.20 (0.52)	2.20 (0.55)		2.20 (0.56)	0.06 (−0.37–0.50)	0.00 (−0.16–0.16)	0.00 (−0.20–0.20)
	CC	2.21 (0.56)	2.24 (0.68)	2.16 (0.56)			−0.04 (−0.24–0.15)	
Physical trait aggression	ICBT	1.78 (0.49)	1.76 (0.48)		1.78 (0.45)	0.12 (−0.31–0.56)	0.03 (−0.20–0.26)	−0.01 (−0.24–0.22)
	CC	1.85 (0.68)	1.83 (0.64)	1.68 (0.56)			0.02 (−0.17–0.21)	
Trait irritability	ICBT	2.65 (0.54)	2.52 (0.55)		2.52 (0.61)	0.36 (−0.09–0.79)	0.23 (0.05–0.41)	0.22 (0.03–0.40)
	CC	2.69 (0.59)	2.71 (0.52)	2.57 (0.65)			−0.04 (−0.27–0.20)	
Mistrust	ICBT	1.81 (0.54)	1.75 (0.49)		1.76 (0.58)	0.53 (0.08–0.97)	0.13 (−0.12–0.38)	0.10 (−0.12–0.32)
	CC	1.95 (0.58)	2.09 (0.76)	1.88 (0.60)			−0.21 (−0.39–0.03)	

Note: SSP, Swedish Universities Scales of Personality; ICBT, Internet cognitive behavior therapy; CC, control condition; Post 2, post-treatment assessment for control condition after having received ICBT.

**Table 4:** Intercorrelations of personality traits and health anxiety at baseline.

	P- factor	HAI	PSTA	STA	SS	LA	E	AS	D	I	SD	VTA	PHTA	TI	M
**HAI**		-													
**Psychic trait anxiety (PSTA)**	**Neuro.**	.29*	-												
**Somatic trait anxiety (STA)**	**Neuro.**	.08	.45**	-											
**Stress susceptibility (SS)**	**Neuro.**	.07	.62**	.48**	-										
**Lack of assertiveness (LA)**	**Neuro.**	.25*	.67**	.31**	.29**	-									
**Embitterment (E)**	**Neuro.**	.20	.58**	.44**	.53**	.35**	-								
**Adventure seeking (AS)**	**Extra.**	.05	.11	.24*	.03	.05	.26*	-							
**Detachment (D)**	**Extra.**	.17	.32**	.16	.33*	.36**	.35**	.01	-						
**Impulsiveness (I)**	**Extra.**	.02	.10	.35**	.23*	−.06	.43**	.58**	.03	-					
**Social desirability (SD)**	**Aggr.**	−.11	−.25*	−.27*	−.25*	−.09	−.46**	−.03	−.35**	−.29**	-				
**Verbal trait aggression (VTA)**	**Aggr.**	.08	.11	.38**	.13	−.26*	.43**	.30**	.13	.43**	−.55**	-			
**Physical trait aggression (PHTA)**	**Aggr.**	−.04	.16	.18	.31**	−.11	.44**	.14	.15	.34**	−.39**	.47**	-		
**Trait irritability (TI)**	**Aggr.**	.19	.41**	.43**	.44**	.15	.53**	.51**	.16	.54**	−.25**	.60**	.41**	-	
**Misstrust (M)**	**Aggr./neuro.**	.16	.53**	.40**	.41**	.33**	.68**	.16	.44**	.17	−.40**	.38**	.33**	.41**	-

Note: * = *p*<.05; ** = *p*<.01; HAI, Health Anxiety Inventory; P, personality; Neuro, neuroticism; Extra., extraversion; Aggr., aggression.

Analyses of within-group changes revealed that participants who received Internet-based CBT significantly reduced their Psychic Trait Anxiety, Somatic Trait Anxiety, Stress Susceptibility, Embitterment, Lack of Assertiveness, and Trait Irritability from pre-to post-treatment (*t*_(39)_ = 2.0–5.1, *p* = .000–.047), while there were no significant changes on the other scales (*t*_(39)_ = 0.0–1.6, *p* = .128–1.00). Moreover, pre-to 6-month follow-up analyses showed that changes in personality were not temporary. The scales on which participants made significant pre-to post-treatment reductions also were significantly lower at 6-month follow-up compared to pre-treatment (*t*_(39)_ = 2.3–5.3, *p* = .000–.025), with the exception of Embitterment (*t*_(39)_ = 0.62, *p* = .540). Finally, there were no pre-treatment to 6-month follow-up changes on the other scales (*t*_(39)_ = 0.0–1.5, *p* = .156–1.00), and participants in the control condition made no significant personality trait changes from pre-to post-treatment (*t*_(40)_ = 0.1–1.5, *p* = .153–.907), with the exception that Mistrust scores significantly increased (*t*_(40)_ = 2.2, *p* = .034). As shown in Table 3, within-group effect sizes were moderately large on most neuroticism-related scales in the ICBT group and small on the other scales. In the control condition, within-group effect sizes were small or negative.

#### Replication of the findings in the control condition after treatment

As the control condition was crossed over to treatment after post-treatment assessment it could be used as a replication sample. Within-group analyses of changes in personality after the control group had received ICBT, i.e. from post-treatment to post-treatment 2, showed that participants made significant reductions on neuroticism-related scales Psychic Trait Anxiety, Somatic Trait Anxiety, and Lack of Assertiveness (*t*_(38)_ = 2.4–4.7, *p* = .000–.023) while changes did not reach statistical significance on the Stress Susceptibility scale (*t*_(38)_ = 2.9, p = .070) and the Embitterment scale (*t*_(38)_ = 1.6, *p* = .125). There were no significant changes on seven of the other eight scales (*t*_(38)_ = 0.1–1.7, *p* = .093–.936) but a significant reduction of scores on the Physical Trait Aggression scale (*t*_(38)_ = 2.8, *p* = .007).

#### Association of changes in personality and health anxiety

To investigate whether changes in personality traits were related to health anxiety improvements change score (pre-to post-treatment) correlations were analyzed. In the sample that first received ICBT, Pearson correlations showed that health anxiety change as measured with the HAI was significantly associated with change in neuroticism-related scales Psychic Trait Anxiety (*r* = .32, *p* = .047), Stress Susceptibility (*r* = .36, *p* = .024), and also with change in Detachment (*r* = .42, *p* = .006) There were no significant associations with health anxiety improvement and changes in the other personality trait scales (*r*s = −.24–.25, *ps* = .116–.948). In the replication sample, i.e. the group that received treatment after 12 weeks of being in the control condition, change in the neuroticism-related scale Somatic Trait Anxiety was significantly correlated with health anxiety improvement measured with the HAI (*r* = .35, *p* = .040). There were no significant associations of changes in the other personality trait scales and health anxiety change in the replication sample (*r* = −.23–.27, *ps* = .13–.80).

## Discussion

This study is to our knowledge the first to investigate the impact of psychological treatment for severe health anxiety on measures of personality traits. In addition, it is one of the first to test the effect of ICBT on personality traits using a randomized controlled design for any anxiety disorder. With experimental control of the independent variable, the results showed that Internet-based ICBT had a significant effect on personality traits related to neuroticism. More specifically, levels of psychic trait anxiety, somatic trait anxiety, stress susceptibility, and embitterment were reduced and the changes were stable to at least 6-month follow-up. The findings of the main analyses were largely validated in the replication sample and improvements in health anxiety were significantly associated with changes in traits of neuroticism.

Although the literature on personality traits and their association with psychiatric disorders is vast, studies investigating the causal effect of treatment on personality for any type of anxiety disorder is scarce. In two studies investigating the effect of pharmacological (alprazolam or diazepam) treatment for panic disorder, Reich, Noyes and co-workers found significant changes in personality traits likely related to neuroticism for patients with large improvement in panic symptoms [52], [53]. The authors concluded that personality assessment is state-dependent, which is a term often used also in the literature on personality and depression based on findings indicating an association between neuroticism and depressive symptoms (e.g. [54], [55]). As the present study showed significant reductions primarily on neuroticism-related scales one could thus argue that traits of neuroticism, but not extraversion or aggression traits, are state-dependent in persons with severe health anxiety. However, considering that participants in the present study had suffered from severe health anxiety for 21 years on average it would not be reasonable to view health anxiety as a “state” affecting personality traits. This trait-like aspect of anxiety disorders was pointed out in a recent review by Brandes and Bienvenu [19] and it is likely that severe health anxiety differs in this regard compared to for example depression, which tends to fluctuate spontaneously to a larger extent [56]. This difference could explain part of the diverging findings of the present study compared to that of Johansson and co-workers investigating personality change in Internet-based CBT for depression [32]. In that study, the personality traits harm avoidance and self-directedness significantly changed after treatment, but no difference in this respect was found between participants who received treatment and the control group on waiting list with access to a discussion forum.

Instead of viewing severe health anxiety as a state affecting personality it is probably more adequate to regard the treatment as having an impact not only on anxiety directly related to health issues, but also on behaviors, emotions and cognitions involved in anxiety and stress in a broader sense, i.e. that which is often referred to as neuroticism. When a person learns to deal with health anxiety through systematically confronting feared events while refraining from safety behaviors, such as searching for information about bodily symptoms on the Internet, it is likely to have some spillover effects on how to handle anxiety and stress in other domains in life. Although neuroticism shares similarities with health anxiety, it is worth noticing that the treatment in this study significantly reduced neuroticism-related traits that were uncorrelated with health anxiety before treatment, such as stress susceptibility.

As neuroticism is a strong predictor of impairment, psychiatric morbidity, quality of life, health service use, and even premature death it has been described as having an enormous significance to public health [57]. A recent study showed that the societal costs of neuroticism are extremely high with per capita excess costs substantially higher than for mental disorders [58] and Lahey [57] has underscored the importance of developing methods targeting neuroticism. Against this background, we regard the findings of the present study as encouraging as they suggest that neuroticism can be reduced through a 12-week Internet-based treatment that was not specifically designed to reduce neuroticism. Therefore, an interesting topic for future research would be to investigate the effect of neuroticism-tailored interventions. This could of course be in the classical form of treatment for persons with high levels of neuroticism, but also as a method to prevent the development of excessive neuroticism.

Two important questions are how similar participants were at baseline compared to the general population and to which extent the treatment changed personality traits in the direction towards it. The baseline estimates on neuroticism trait scales showed that these were up to one standard deviation higher in the severe health anxiety sample compared to data sampled from the general population [20]. This pattern was however not present on extraversion or aggression trait scales were baseline estimates in the severe health anxiety sample was similar or slightly lower compared to the general population. This could probably to some extent explain the findings that there were no significant differences in changes on these non-neuroticism-related trait scales. That is, if the participants were similar to the general population on these traits at baseline, there is no obvious reason why a treatment aimed at reducing clearly exaggerated anxiety but not focusing on aggression or extraversion would affect the latter factors. This is consistent with the literature on personality change after CBT for eating disorders where some factors, such as harm avoidance, have been shown to be more susceptible to change than others [30]. On the traits where Internet-based CBT did cause a change, the change was in the direction towards the mean of the general population, i.e. after treatment patients were more similar to the average person in terms of psychic and somatic trait anxiety, stress susceptibility, embitterment and lack of assertiveness.

The most important strength of the present study was the randomized controlled design making it possible to infer that the personality trait changes were caused by the treatment and not merely reflecting the passage of time. This interpretation is supported by the fact that similar changes were observed in the control group only when they were switched over to active treatment. To our knowledge, this has not been demonstrated before when it comes to any form of psychological treatment for severe health anxiety. Other strengths of the study were the low attrition rates and the well-validated measures of personality traits and health anxiety. As for limitations, one central was that we did not conduct any type of objective behavioral test to check if personality changes as assessed with SSP were related to observable behaviors. Previous studies on the SSP have however demonstrated that scores are related to observer assessments of psychiatric symptoms [59]. Also, as participants in the control group were crossed over to ICBT after post-treatment between-group comparisons could not be made after this time point. Another limitation was that the design makes it impossible to say if exposure-based CBT is necessary for achieving the changes in personality observed in the present study. A venue for future research is to investigate if similar effects can be seen in other treatments such as pharmacological or psychodynamic therapy for severe health anxiety. It would also be valuable to investigate whether personality traits are prognostic factors for treatment outcome, i.e. whether patients with certain traits are more likely to respond to treatment.

In spite of these limitations we regard the present study as important as it shows for the first time that 12 weeks of ICBT for severe health anxiety has a significant and stable impact on personality traits of neuroticism. After treatment, the personality profile of persons with severe health anxiety is more similar to that of the general population.
